# Endocrine Therapy Initiation among Older Women with Ductal Carcinoma In Situ

**DOI:** 10.1155/2017/6091709

**Published:** 2017-09-13

**Authors:** Chelsea Anderson, Aaron N. Winn, Stacie B. Dusetzina, Hazel B. Nichols

**Affiliations:** ^1^Department of Epidemiology, Gillings School of Global Public Health, University of North Carolina at Chapel Hill, Chapel Hill, NC, USA; ^2^Department of Health Policy and Management, Gillings School of Global Public Health, University of North Carolina at Chapel Hill, Chapel Hill, NC, USA; ^3^Division of Pharmaceutical Outcomes and Policy, UNC Eshelman School of Pharmacy, University of North Carolina at Chapel Hill, Chapel Hill, NC, USA; ^4^UNC Lineberger Comprehensive Cancer Center, Chapel Hill, NC, USA

## Abstract

**Background:**

Although treatment of ductal carcinoma in situ (DCIS) is controversial, national guidelines recommend considering endocrine therapy for women with estrogen receptor- (ER-) positive DCIS or those undergoing breast conserving surgery (BCS) without radiation. We evaluated uptake and predictors of endocrine therapy use among older women with DCIS.

**Methods:**

In the SEER-Medicare database, we identified women aged 65+ years diagnosed with DCIS during 2007–2011. We evaluated demographic, tumor, and treatment characteristics associated with endocrine therapy initiation.

**Results:**

Among 2,945 women with DCIS, 41% initiated endocrine therapy (66% tamoxifen, 34% aromatase inhibitors). Initiation was more common among women with ER-positive than ER-negative DCIS (48% versus 16%; HR = 3.75, 95% CI: 2.91–4.83); 28% of women with unknown ER status initiated endocrine therapy. Initiation was less common after BCS alone compared to BCS with radiation (32% versus 50%; HR = 0.69, 95% CI: 0.59–0.80).

**Conclusions:**

Less than half of older women with DCIS initiate endocrine therapy to prevent second breast cancers. Our findings suggest use was more common, but not exclusive, among women with ER-positive DCIS, but not among women who underwent BCS alone. Endocrine therapy should be targeted toward patients most likely to benefit from its use.

## 1. Introduction

Treatment strategies for ductal carcinoma in situ (DCIS), a stage 0 breast cancer frequently detected by mammogram, are controversial. DCIS accounts for >20% of all breast cancer diagnoses in the United States, with over 60,000 incident cases each year [1]. Prognosis for DCIS is excellent, and 10-year survival exceeds 97% [2]. However, the risk of developing invasive breast cancer is elevated among women with a DCIS diagnosis relative to women in the general population [3]. Breast conserving surgery (BCS) with radiation or mastectomy is currently standard of care therapies for DCIS [4]. Endocrine therapy after DCIS is widely recognized to reduce second breast cancer events and future invasive disease, with randomized clinical trials suggesting a risk reduction of 29–37% [5, 6].

Tamoxifen was approved by the US Food and Drug Administration (FDA) as endocrine therapy for DCIS in 2000, and recent trials have reported promising results for other endocrine agents, including the aromatase inhibitor anastrozole [7, 8]. National Comprehensive Cancer Network (NCCN) guidelines currently recommend that postmenopausal women with estrogen receptor- (ER-) positive DCIS consider using tamoxifen or an aromatase inhibitor to reduce the risk of a second breast cancer, while noting that the benefit of endocrine therapy for women with ER-negative DCIS is uncertain. Current guidelines also recommend consideration of endocrine therapy for DCIS patients who undergo BCS without radiation [4]. Prior reports suggest marked variation in endocrine therapy initiation among women with a DCIS diagnosis, with estimates ranging from less than 20% to greater than 70% across previous studies [9–18]. Though patient demographics, tumor characteristics, and concurrent treatments may influence the decision to initiate endocrine therapy, much of the variability in use remains unexplained.

Using the SEER-Medicare database, we estimated the proportion of women aged 65 and older who receive endocrine therapy in the year following DCIS and evaluated factors associated with endocrine therapy initiation, including surgery, radiation, and other patient and tumor characteristics.

## 2. Materials and Methods

### 2.1. Study Sample and Data Sources

We used the Surveillance, Epidemiology, and End Results (SEER) Medicare linked data to evaluate factors associated with endocrine therapy use among women with DCIS. The SEER-Medicare data combine data from cancer registries covering 28% of the US population and fee-for-service Medicare administrative claims for individuals aged 65 years and older who are diagnosed with cancer. We identified 3,047 women who were diagnosed with DCIS during 2007–2011, did not have a previous cancer diagnosis, and were enrolled in fee-for-service Medicare Parts A (hospital), B (outpatient), and D (prescription drug) plans at diagnosis. We excluded those without documented breast surgery (breast conserving surgery or mastectomy) (*n* = 102).

We classified women as endocrine therapy users if they filled any prescription for endocrine therapy (tamoxifen, anastrozole, exemestane, and letrozole) during the year after breast surgery. Additional variables of interest included age, year of diagnosis, race, tumor characteristics (estrogen receptor status, grade, tumor size, and comedo status), treatment received after diagnosis (breast conserving surgery, mastectomy, and radiation therapy), and geographic area.

### 2.2. Statistical Analysis

Cox proportional hazard models were used to estimate hazard ratios (HR) and 95% confidence intervals (CI) for endocrine therapy initiation. Person-time was calculated beginning at the date of breast surgery. Individuals were censored due to death or disenrollment in a fee-for-service plan. All variables were first evaluated in models adjusted for age at diagnosis. Multivariable models were then estimated with adjustment for characteristics associated with endocrine therapy use in age-adjusted models. Women with missing data for adjustment variables were excluded from multivariable models.

The study protocol was considered exempt by the University of North Carolina at Chapel Hill's Institutional Review Board.

## 3. Results and Discussion

### 3.1. Results

A total of 2,945 women with a DCIS diagnosis contributed to our analysis. The average age at diagnosis was 72.3 years (SD = 5.6) among women who initiated endocrine therapy and 74.5 years (SD = 6.6) among women who did not. Most women were white (84%) and had ER-positive tumors (72%) that were less than 40 mm in size (69%) and were either grade 2 (38%) or grade 3 (36%). Breast conserving surgery with radiation was the most common treatment (52%), followed by breast conserving therapy without radiation (24%) and mastectomy (24%). Overall, 41% of women initiated endocrine therapy. Among endocrine therapy users, 66% used tamoxifen, and 34% used aromatase inhibitors (Table 1).

**Figure 1:**
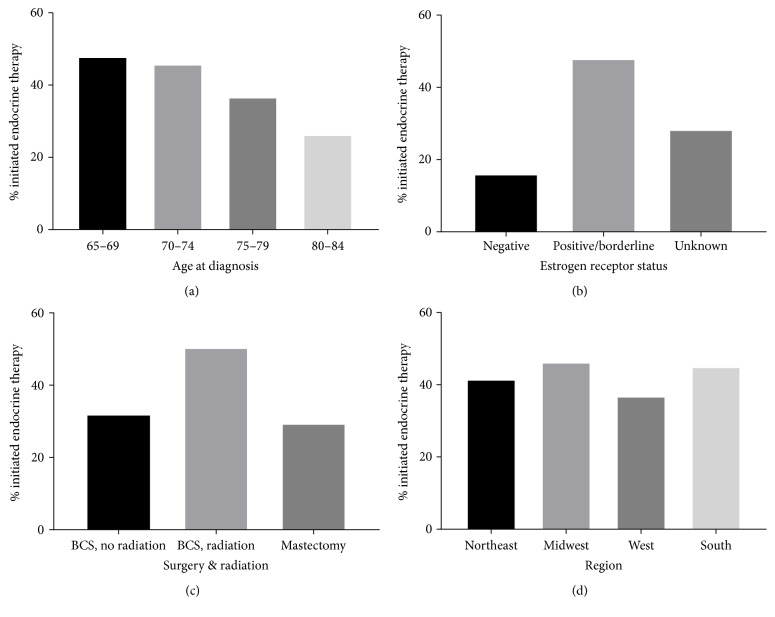
Endocrine therapy initiation among women aged 65+ years diagnosed with ductal carcinoma in situ during 2007–2011 in SEER-Medicare according to (a) age at diagnosis, (b) estrogen receptor status, (c) surgery and radiation, and (d) region.

The proportion of patients who initiated endocrine therapy according to age at diagnosis, ER status, surgery and radiation, and geographic region is shown in Figure 1. Likelihood of initiation decreased with increasing age at diagnosis, with 48% among those aged 65–69 years and 26% among those aged 80–84 years. Among women with ER-positive DCIS, 48% initiated endocrine therapy, compared to 28% and 16% among those with unknown ER status and ER-negative status, respectively. Initiation was more common among those treated with BCS and radiation (50%) than among those treated with either mastectomy (29%) or BCS without radiation (32%). Across geographic regions, the proportion that initiated endocrine therapy was highest among those in the Midwest (46%) and lowest among those in the West (37%).

In multivariable analyses, endocrine therapy use was inversely associated with age. Relative to women aged 65–69, those aged 75–79 (HR = 0.74; 95% CI: 0.63, 0.87) and 80+ (HR = 0.55; 95% CI: 0.45, 0.66) were less likely to initiate endocrine therapy (Table 1). Region was also associated with endocrine therapy use; compared to women from the South, women from the Northeast (HR = 0.77; 95% CI: 0.65, 0.92) and West (HR = 0.67; 95% CI: 0.57, 0.78) were less likely to use endocrine therapy.

Initiation of endocrine therapy was more common among women with ER-positive tumors (HR = 3.75; 95% CI: 2.91–4.83) and women with tumors of unknown ER status (HR = 2.12; 95% CI: 1.56–2.88) than among those with ER-negative tumors. Treatment was also associated with likelihood of endocrine therapy use. Relative to women who underwent radiation and BCS, those who underwent mastectomy (HR = 0.59; 95% CI: 0.51, 0.69) or BCS alone (HR = 0.69; 95% CI: 0.59, 0.80) were less likely to use endocrine therapy. Tumor size, grade, and comedo status did not appear to be strongly associated with endocrine therapy initiation.

### 3.2. Discussion

Randomized clinical trials have demonstrated a reduction in risk of second breast cancers with use of endocrine therapy following DCIS [5–8], particularly for women with ER-positive tumors [19]. Current NCCN guidelines recommend consideration of a 5-year course of tamoxifen or aromatase inhibitors for postmenopausal women who have ER-positive DCIS or are treated with BCS without radiation [4], yet most recent reports have suggested a relatively low uptake of endocrine therapy among DCIS patients [10–15, 18, 20]. In our analysis, 41% of women with a DCIS diagnosis at the age of 65 and older initiated endocrine therapy. In addition to ER-positive DCIS, other characteristics associated with endocrine therapy initiation included younger age at diagnosis and treatment with BCS and radiation. Tumor characteristics, such as size, grade, and comedo status, were not strongly associated with initiation in our study.

Our results are largely in agreement with a recent report by Zhao and colleagues that evaluated endocrine therapy use among women with ER-positive DCIS using combined data from SEER-Medicare and the Texas Cancer Registry [20]. However, in the current study, we also included women with ER-negative DCIS and those with unknown ER status. Interestingly, we found that 16% of those with ER-negative tumors initiated endocrine therapy, despite current guidelines citing an unclear benefit in this group [4]. We also demonstrated that an estimated 14% of older women with DCIS may not know the ER status of their tumor at the time of ET initiation, and over one-quarter of these women initiated endocrine therapy in the year following diagnosis. These findings are unlikely to be explained by progesterone receptor (PR) status, as only 5% of women with unknown ER status or ER-negative DCIS in our sample had PR-positive DCIS. Women with DCIS are included in some chemoprevention trials [21, 22], where use is similarly not related to known ER status of a primary tumor. Our findings may suggest that, similar to the chemoprevention context, it is perceived vulnerability to a second breast cancer [23], rather than specific tumor characteristics, that motivates endocrine therapy initiation among older women with DCIS [10].

In our study, we found that approximately 34% of those who initiated endocrine therapy used an aromatase inhibitor, a somewhat higher proportion than that reported by Zhao et al. (24%) [20]. Though tamoxifen has been approved as adjuvant therapy for DCIS since 2000, aromatase inhibitors have not been FDA-approved for this indication, suggesting considerable off-label use. Recent reports from the IBIS-II DCIS and NSABP B-35 trials have demonstrated a benefit of aromatase inhibitors similar to that of tamoxifen for postmenopausal women with DCIS [7, 8]. However, tamoxifen and aromatase inhibitors have potential side effects that should be considered before initiating treatment in women without strong evidence of benefit. Tamoxifen, for example, is associated with an elevated risk of stroke and venous thromboembolism [24, 25], while aromatase inhibitors may increase risk of osteoporosis [26], all concerns which may be especially relevant for older women. The risk of these adverse outcomes, combined with the lack of proven survival benefit among women with DCIS [27], may explain the relatively low uptake of endocrine therapy (41%) in our cohort of women aged 65 years and older.

We also found that initiation was more common among women who underwent BCS and radiation compared to those who underwent BCS without radiation. This finding contrasts with NCCN guidelines to consider endocrine therapy specifically among women who undergo excision alone [4] but is consistent with prior reports [10, 11, 16, 18]. In our prior work, we identified women in North Carolina with a DCIS diagnosis during 2006–2010 and reported that those who underwent BCS without radiation were 0.63 (95% CI: 0.50–0.78) times as likely to receive adjuvant endocrine therapy as those who underwent BCS with radiation [18]. A similar finding was reported by Nichols et al. in a Seattle-based integrated health setting (RR = 0.46; 95% CI: 0.25–0.84) [10]. Of note, these studies were set within limited geographic areas but were not restricted to women aged 65 years and older. Taken together, findings from these prior reports and from the current study suggest that the association between more extensive initial treatment and endocrine therapy initiation persists across age groups and geographic regions. Women who opt for radiation, in addition to BCS, may have a higher perceived risk of recurrence or invasive breast cancer than those who undergo BCS alone, a characteristic which may also explain their higher likelihood of endocrine therapy initiation.

Strengths of the current study include the large sample size and availability of endocrine therapy from Medicare claims, an important treatment consideration that is not available in the SEER registry data alone. However, some limitations should also be considered. Because only women aged 65 years and older are included in the SEER-Medicare database, our findings may not generalize to younger women. Additionally, we were unable to categorize women according to their risk profile or contraindications for using tamoxifen versus aromatase inhibitors, because key aspects of medical history (e.g., prior hysterectomy) prior to Medicare enrollment are not available in the SEER-Medicare database. We were also unable to assess the influence of other patient characteristics, such as family history,* BRCA* status, or perceived risk of a second breast event.

## 4. Conclusions

Endocrine therapy initiation is a critical treatment consideration in the context of current debates over the appropriate clinical care for women with DCIS. This study highlights characteristics associated with endocrine therapy use among women aged 65 and older with a DCIS diagnosis, particularly ER-positive tumors and receipt of breast conserving surgery with radiation. Endocrine therapy should be targeted toward patients for whom the benefits are most likely to outweigh the risks.

## Figures and Tables

**Table 1:** Characteristics among women with a DCIS diagnosis in relation to use of endocrine therapy, SEER-Medicare, 2007–2011.

	Endocrine therapy^a^	No endocrine therapy	HR (95% CI)^b^	HR (95% CI)^c^
	*N*	*N*		
Total	1,205	1,740		
*Age at diagnosis*, years				
65–69	455	494	1	1
70–74	374	442	0.94 (0.82, 1.08)	0.94 (0.82, 1.07)
75–79	230	396	0.73 (0.63, 0.86)	0.74 (0.63, 0.87)
80-84	146	408	0.49 (0.41, 0.60)	0.55 (0.45, 0.66)
Continuous age at diagnosis (SD)	72.3	74.5	0.96 (0.95, 0.97)	0.97 (0.96, 0.98)
*Calendar year of diagnosis*				
2007	242	324	1	1
2008	244	362	0.92 (0.77, 1.10)	1.03 (0.92, 1.16)
2009	233	370	0.87 (0.72, 1.04)	1.07 (0.95, 1.20)
2010	219	333	0.88 (0.73, 1.06)	1.07 (0.95, 1.21)
2011	267	351	0.99 (0.83, 1.18)	1.04 (0.93, 1.16)
*Race*				
White	1,027	1,458	1	1
Black	96	139	0.97 (0.79, 1.20)	0.87 (0.70, 1.08)
Asian, other or unknown	82	143	0.83 (0.66, 1.04)	1.00 (0.79, 1.27)
*Geographic area*				
Northeast	266	374	0.88 (0.75, 1.04)	0.77 (0.65, 0.92)
Midwest	181	210	1.07 (0.89, 1.29)	0.93 (0.77, 1.12)
West	471	806	0.74 (0.64, 0.85)	0.67 (0.57, 0.78)
South	287	350	1	1
*Estrogen receptor (ER) status*				
ER negative	64	334	1	1
ER positive/borderline	1,023	1,108	3.70 (2.87, 4.76)	3.75 (2.91, 4.83)
ER unknown	118	298	2.02 (1.49, 2.74)	2.12 (1.56, 2.88)
*Surgery & radiation*				
Breast conserving surgery, no radiation	224	475	0.69 (0.59, 0.80)	0.69 (0.59, 0.80)
Breast conserving surgery, radiation	767	753	1	1
Mastectomy	214	512	0.57 (0.49, 0.66)	0.59 (0.51, 0.69)
*Grade*				
1	182	236	1	1
2	499	613	0.95 (0.80, 1.13)	0.95 (0.80, 1.13)
3	368	684	0.70 (0.58, 0.83)	0.88 (0.73, 1.06)
Missing	156	207	0.92 (0.74, 1.13)	0.99 (0.80, 1.23)
*Tumor size, mm*				
≤15	623	843	1	1
16–40	206	351	0.82 (0.70, 0.95)	0.92 (0.79, 1.08)
>40	80	145	0.80 (0.63, 1.00)	0.99 (0.78, 1.26)
Unknown	296	401	0.98 (0.85, 1.12)	1.02 (0.89, 1.18)
*Comedo status*				
No	1,040	1,460	1	1
Yes	165	280	0.84 (0.71, 0.99)	1.01 (0.86, 1.19)

^a^797 tamoxifen users plus 408 aromatase inhibitor users; ^b^adjusted for age at diagnosis; ^c^adjusted for age at diagnosis, race, ER status, surgery and radiation, and geographic area.
